# Comparing magnetic resonance liver fat fraction measurements with histology in fibrosis: the difference between proton density fat fraction and tissue mass fat fraction

**DOI:** 10.1007/s10334-022-01052-0

**Published:** 2022-12-20

**Authors:** Stephen James Bawden, Caroline Hoad, Philip Kaye, Mary Stephenson, Grace Dolman, Martin W. James, Emilie Wilkes, Andrew Austin, Indra Neil Guha, Susan Francis, Penny Gowland, Guruprasad P. Aithal

**Affiliations:** 1 Nottingham Digestive Diseases Centre, NIHR Nottingham Biomedical Research Centre at the Nottingham University Hospitals NHS Trust and University of Nottingham, Nottingham, NG7 2RD UK; 2 Sir Peter Mansfield Imaging Centre, SPMIC, University Park, Physics and Astronomy, University of Nottingham, Nottingham, UK; 3 Department of Cellular Pathology, Nottingham University Hospitals NHS Trust, Nottingham, UK; 4 Clinical Imaging Research Centre (CIRC), Agency for Science, Technology and Research (A*STAR), Singapore, Singapore; 5 Derby Royal Hospital, Derby, UK

**Keywords:** Non-alcoholic fatty liver disease, Fatty liver, Fibrosis, Liver cirrhosis, Liver diseases

## Abstract

**Objective:**

Magnetic resonance spectroscopy (MRS) provides a powerful method of measuring fat fraction. However, previous studies have shown that MRS results give lower values compared with visual estimates from biopsies in fibrotic livers. This study investigated these discrepancies and considered whether a tissue water content correction, as assessed by MRI relaxometry, could provide better agreement.

**Materials and methods:**

110 patients were scanned in a 1.5 T Philips scanner and biopsies were obtained. Multiple echo MRS (30 × 30 ×  30 mm volume) was used to determine Proton Density Fat Fraction (PDFF). Biopsies were assessed by visual assessment for fibrosis and steatosis grading. Digital image analysis (DIA) was also used to quantify fat fraction within tissue samples. T_1_ relaxation times were then used to estimate tissue water content to correct PDFF for confounding factors.

**Results:**

PDFF values across the four visually assessed steatosis grades were significantly less in the higher fibrosis group (F3–F4) compared to the lower fibrosis group (F0–F2). The slope of the linear regression of PDFF vs DIA fat fraction was ~ 1 in the low fibrosis group and 0.77 in the high fibrosis group. Correcting for water content based on T_1_ increased the gradient but it did not reach unity.

**Discussion:**

In fibrotic livers, PDFF underestimated fat fraction compared to DIA methods. Values were improved by applying a water content correction, but fat fractions were still underestimated.

**Supplementary Information:**

The online version contains supplementary material available at 10.1007/s10334-022-01052-0.

## Introduction

The prevalence of Hepatic metabolic disorders, type II diabetes and non-alcoholic steatohepatitis (NASH) as well as non-alcoholic fatty liver disease (NAFLD) [1, 2] is increasing globally [3, 4]. These conditions are associated with a spectrum of histological manifestations from steatosis to steatohepatitis and the consequent development of fibrosis and cirrhosis. As such, there is an increasing requirement for an effective method of assessing liver fat fraction in progressive liver disease. The clinical gold standard for measuring hepatic fat content is histological assessment, which is an expensive and invasive procedure and subject to sampling error [5–7]. Conventionally, steatosis is graded by qualitative visual assessment (VA) of the fractional area of fat vacuoles within the total tissue (% hepatocytes) [8]. Alternatively, fractional fat content within biopsies can be automatically quantified using digital image analysis (DIA) of stained samples [9, 10], but many biopsies performed in clinical practice fail to meet recommended standards for such assessment [11]. In either case, histological methods seek to determine the fractional content of fat within a tissue sample (tissue mass fat fraction, TMFF).

Magnetic resonance (MR) methods, such magnetic resonance spectroscopy (MRS) and chemical shift encoded MRI (CSE-MRI), have recently been used to provide a quantitative non-invasive method of measuring hepatic fat content [12, 13]. They have been well validated [14–16] and used in multiple studies to investigate NAFLD and related metabolic disorders [17, 18]. MRS can also offer unique insights into lipid composition [19, 20].

An important point which is sometimes overlooked clinically is that, in actual fact, both MRS and CSE-MRI calculate the proton density fat fraction (PDFF) rather than the true tissue mass fat fraction (TMFF) which is ultimately the clinical endpoint of interest [14]. Whereas TMFF measures the fat-to-tissue mass ratio, PDFF is a measure of the fat-to-water MR signal ratio. It is therefore important to note that MR is measuring a different (though related) endpoint compared to histological assessments despite the widespread and often unhelpful adoption of the term ‘fat fraction’ in both cases. Whilst PDFF is a powerful non-invasive measurement related to liver disease, there may be situations where TMFF is more applicable. Furthermore, using the two measurements interchangeably could lead to potential MR confounders in PDFF compared to TMFF being neglected. For example, Hamilton et al. [20] investigated the effects of PRESS v STEAM MRS localization and found that these measurement techniques can influence T_2_ due to J-coupling effects and lead to variation in fat fraction values.

Attempts have been made to convert PDFF to TMFF by including factors based on literature defined proton densities, mass densities and tissue water content [21]. Interestingly, in healthy livers, these approaches result in a close correspondence between PDFF and TMFF which can propagate the mistaken thought that both techniques are measuring the same thing. However, the conversion may not hold in liver disease where values of tissue water content may vary [22, 23]. This has important implications for trials across different liver etiologies or when studying disease progression and needs to be considered fully before widespread adoption of MR fat fraction measurements in a clinical setting.

One example of this is seen in recent work that has suggested that MR estimates of hepatic fat fraction are reduced compared to histology in fibrotic patients [15, 24]. Other studies have shown conflicting results [25, 26] leading to confusion on the impact of fibrosis in MR derived fat fractions. None of these studies, however, used an independent non-MR based objective method to estimate tissue fat fraction (e.g. automated digital image analysis, DIA), which would help to rule out subjective variation, nor did they consider the effects of non-fat related physiological changes (e.g. tissue water content) on MR measurements.

In this study, MR measurements of fat fraction were compared to objective histological assessment (DIA) in healthy and fibrotic patients. Tissue water content was then explored as a potential source of systematic error in MR estimates of fat fraction in the fibrotic group and a novel T_1_ based estimate of free water content was developed to correct this.

## Materials and methods

This study was undertaken with patients from Nottingham University Hospitals NHS Trust and Derby Teaching Hospitals NHS Foundation Trust between May 2009 and September 2012 and was approved by the Nottingham Research Ethics Committee with all patients giving written, informed consent. Patients who had undergone liver biopsy were recruited as part of a wider study investigating MR changes in liver fibrosis [27]. Inclusion criteria were a liver biopsy length > 2.5 cm and time between biopsy and MRS < 3 months. Patients were excluded for contraindications for MRI scanning (e.g. pacemakers, aneurysm clips etc.). 110 subjects were scanned in total on a single occasion in a 1.5 T Philips Achieva scanner (Best, Netherlands) with a body transmit and 5 element SENSE receive coil.

### ^1^H MRS measurement of liver fat content

^1^H MRS was acquired from a 30 × 30 × 30 mm^3^ voxel positioned within the lower right lobe of the liver in the same region as biopsies [27] and away from major blood vessels. Point resolved spectroscopy (PRESS) localization was applied with respiratory triggering at 4 different echo times (TE) to correct for T_2_ relaxation (whilst previous work suggests STEAM localization reduces errors in T_2_ and fat fraction estimations, PRESS was used in the present study because it is less sensitive to motion). 16 spectra were averaged at the shortest TE (30 ms) for greatest fat-to-water signal to noise ratio (SNR) and 8 spectra were averaged for subsequent echo times (40, 60 and 80 ms). A minimum TR of 3000 ms was fixed to allow full longitudinal recovery of all spectra. A respiratory triggered sequence was chosen due to the large range in patient conditions and effects of breathing.

**Fig. 1:**
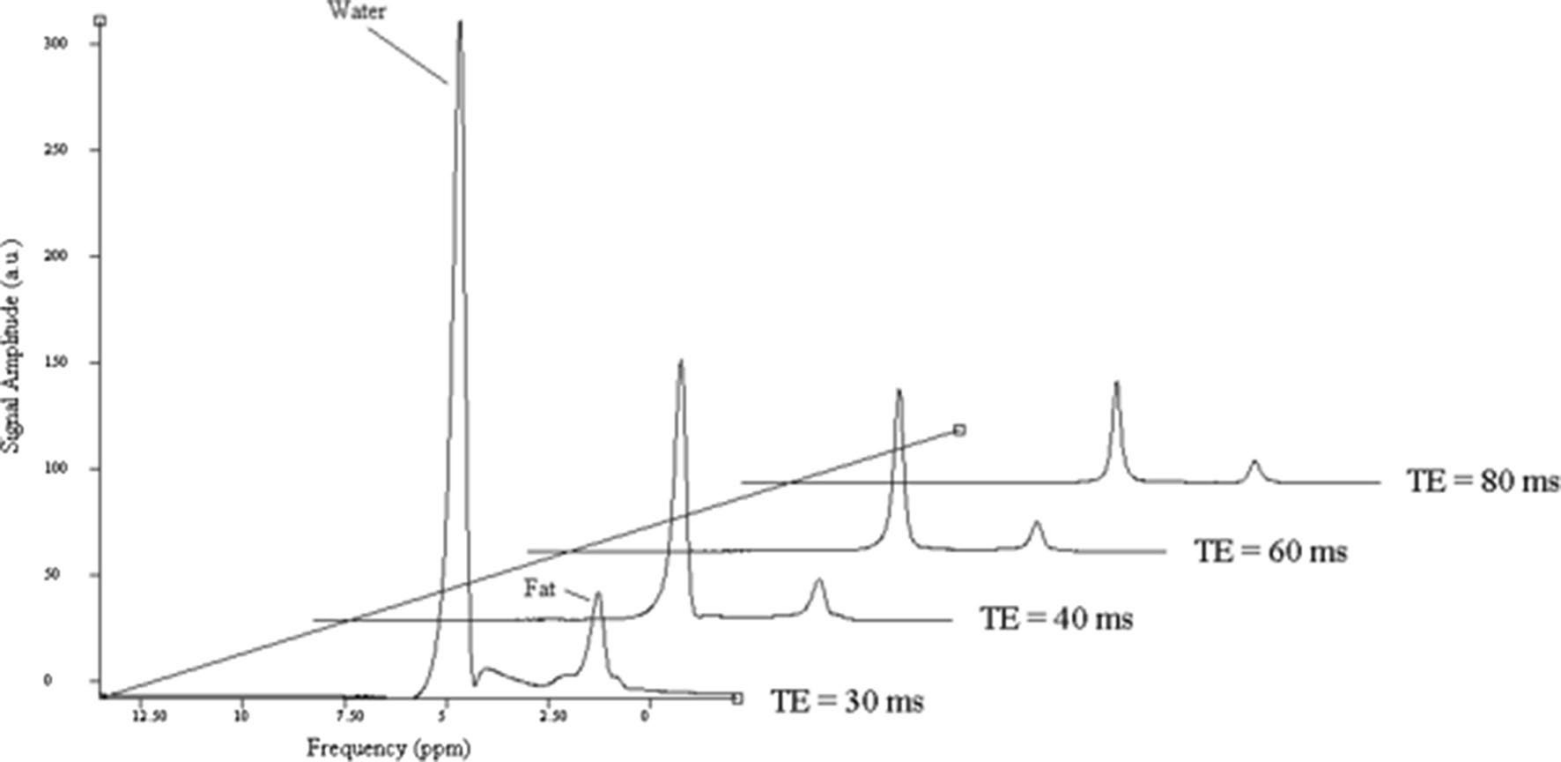
Example spectra from one patient showing the average at each echo time (TE = 30, 40, 60 and 80 ms). The ratio of fat-to-water peak is used to determine fat fraction

Spectra were analyzed using Java-based Magnetic Resonance User Interface (jMRUI) [28]. Individual spectra were phase corrected and frequency aligned before averaging across each TE. The area under the main water and lipid peaks were calculated by integrating Lorentzian peaks fits using AMARES within jMRUI, and the value at each echo time fitted to a mono-exponential decay curve to calculate the T_2_ relaxation time. T_2_ corrected signals where then used to determine PDFF and tissue mass fat fraction (TMFF_MR_) (Fig. 1).

### MRI measurement of ${T}_{1}$ relaxation time

T_1_ was measured using a respiratory triggered inversion recovery EPI sequence covering the whole liver with fat suppression to remove lipid T_1_ effects. Ten inversion times were acquired and data were fitted as described previously [27].

### Pathologist's visual assessment (VA)

Liver biopsies were stained with Hematoxylin–eosin (H&E). Only samples with a minimum biopsy length of 2.5 cm were included. The degree of steatosis and fibrosis was graded by a pathologist blinded to the MRS data according to the NASH-Clinical research Network (CRN) scoring [29]. CRN steatosis grading was as follows: percentage of parenchymal steatosis involvement < 5% S = 0, 5%-33% S = 1, > 33%-66% S = 2, > 66% S = 3. CRN Fibrosis grading was divided as follows: None F = 0, Perisinusoidal or periportal F = 1, Perisinusoidal and periportal F = 2, Bridging fibrosis F = 3, Cirrhosis F = 4. The same histopathologist also made a visual estimation of the percentage fat fraction. Inflammation of the biopsy was determined as previously described [27] and etiology defined (alcohol liver disease, hemochromatosis, hepatitis B and C, NAFLD, normal or other).

### Digital image analysis (DIA)

Automated digital image analysis was also performed on biopsies. Samples were scanned using a Nanozoomer whole slide scanning system (Hamamatsu, Japan) at a magnification of × 20. Images were imported as TIFF files, with a magnification of × 5. Slides were processed using iTEM^©^ software (Olympus, Germany). An in-house macro was used which required minimal user interaction and had fixed image thresholding settings. The macro automatically identified the outline of the biopsy which was confirmed by the user. The software calculated the area in pixels. If the biopsy was fragmented, or there was more than one core, the process was repeated until all areas of the biopsy were selected. The program measured white pixels within the selected area and designated these as steatosis. The final DIA fat fraction was determined as the area of white pixels as a fraction of the whole biopsy.

Initially, comparisons of the DIA area fat fraction (DIAFF) with PDFF were made without any conversion or correction to mass fraction. The DIA fat fraction was then used to calculate a mass fat fraction estimate. To account for the conversion of area-density to volume-density, a circle/square $\left( {\pi r^{2} /2r^{2} } \right)$ to sphere/cube $\left( {[4/3]\pi r^{3} /2r^{3} } \right)$ ratio was applied (assuming roughly spherical lipid deposition and that cross-sectional density is similar to the volume density). Finally, the fat-to-water mass density ratio ($0.9$) was used to convert to mass fat fraction (TMFF_DIA_) and compared with the MR derived tissue mass fat fraction (TMFF_MR_).

### Modelling the effects of tissue water content on PDFF

Initially, PDFF was calculated as $S_{F} /\left( {S_{F} + S_{W} } \right)$ (where ${S}_{F}$ and ${S}_{W}$ are the T_2_ corrected signals from fat and water, respectively) and compared with DIAFF. The PDFF was then used to calculate a mass fat fraction estimate TMFF_MR_ as outlined below.

The MR signal ($S$) from water or fat in the tissue depends on the amount of each within the acquisition volume and their respective proton densities $\rho$ (mmol/l) as follows:

1 $$S = S_{H} \rho \frac{M}{D}$$

where ${S}_{H}$ is the signal from 1 mmol of hydrogen; and $M$ and $D$ are the mass and density respectively. From this, the ratio of fat-to-water MR signals (${R}_{s}$) becomes:

2 $$R_{s} = \frac{{M_{F} \rho_{F} D_{W} }}{{M_{W} \rho_{W} D_{F} }} = 0.7 R_{M}$$

where subscripts $F$ and $W$ denote the fat and water values respectively, ${R}_{M}$ is the fat-to-water mass ratio in the volume, ${\rho }_{F}$ = 70.35 mmol/l, ${\rho }_{W}$= 111.11 mmol/l, ${D}_{F}$= 0.9 g/ml and ${D}_{W}$= 1 g/ml [14].

**Fig. 2:**
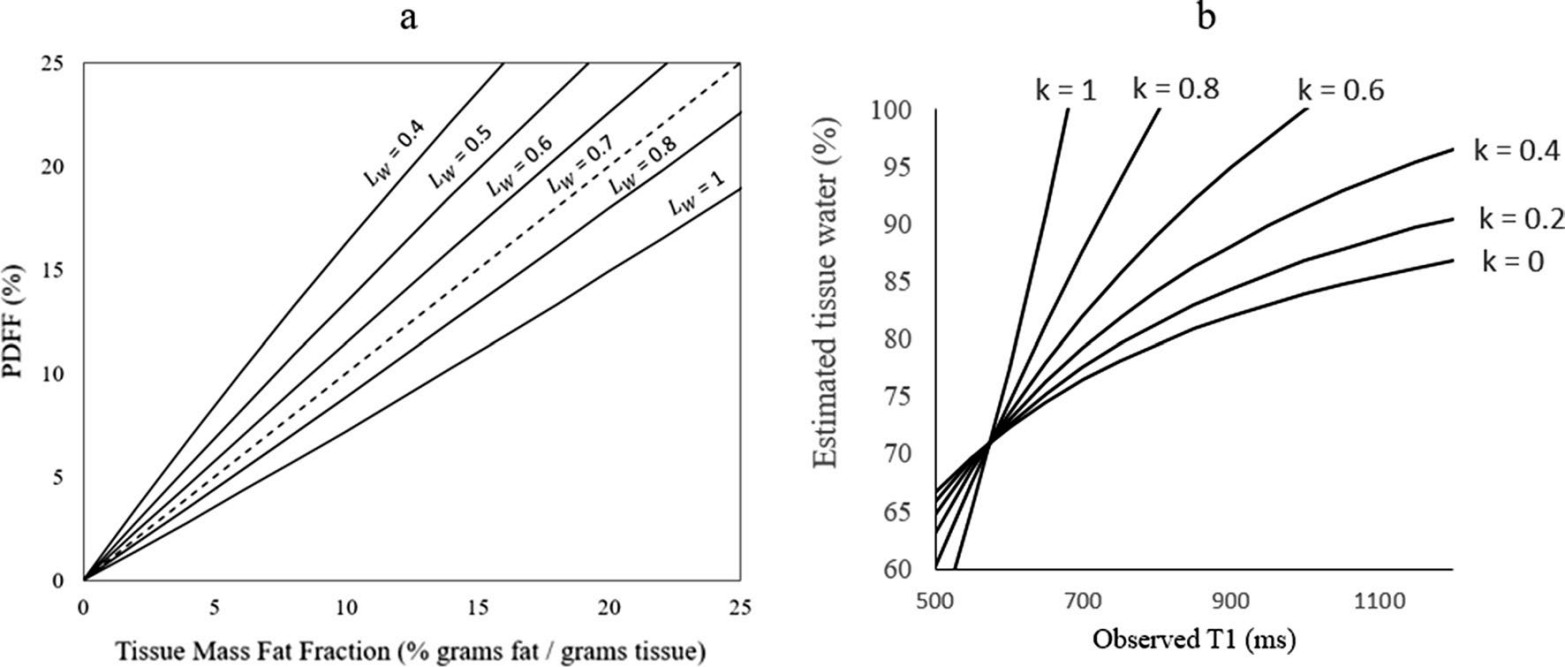
Model of Proton Density Fat Fraction as a function of Tissue Mass Fat Fraction for a range of tissue water content values (${\mathrm{T}}_{\mathrm{W}}$). **a** Model of tissue water content fraction as a function of observed ${\mathrm{T}}_{1}$ for a range of proportionality constant $k$ (representing the fraction of change in tissue water coming from non-water compartments)

The tissue mass fat fraction (TMFF) measures the fraction of fat to *lean tissue (*which does not just include water and fat) and so can be calculated as follows:

3 $$TMFF = \frac{{R_{M} L_{W} }}{{R_{M} L_{W} + 1}}$$

where ${L}_{W}$ is the tissue water fraction. In this study, Eq. 3 was used to model the relationship between PDFF and TMFF and investigate the effects of tissue water content on PDFF (Fig. 2a). The tissue water fraction in healthy livers has previously been defined as ${T}_{w}$ = 0.711 [18] which, from Eqs. 2 and 3, results in $PDFF\approx TMFF$.

### Modelling the relationship between tissue water content and measured T_1_

MR techniques are only sensitive to signal from the free water, but tissue also contains bound water which has a fast MR signal decay rate. Whilst bound water cannot be detected in MR due to this rapid decay, it can affect T_1_ due to the fast exchange between the two water pools. Assuming fast exchange, observed fat-suppressed T_1_ from tissue is given by the weighted combination of the free and bound water T_1_ values (${T}_{1,F}$ and ${T}_{1,B}$ respectively) as follows:

4 $$\frac{1}{{T_{1,O} }} = \frac{{W_{F} }}{{T_{1,F} }} + \frac{{W_{B} }}{{T_{1,B} }} = \frac{{W_{F} }}{{T_{1,F} }} + \frac{{\left( {1 - W_{F} } \right)}}{{T_{1,B} }}$$

where ${W}_{F}$ and ${W}_{B}$ are the free and bound water fractions in water containing tissue, respectively [30]. Using previously reported values of ${T}_{1,F}$ and ${T}_{1,B}$ [31] the observed ${T}_{1,O}$ can therefore be used to estimate the fraction of free water ${W}_{F}$.

Furthermore, using a three compartment model where ${L}_{W}$, ${L}_{B}$ and ${L}_{N}$ represent the free-water, bound-water and non-water liver tissue fractions, respectively (${L}_{W}+{L}_{B}+{L}_{N}=1$), any changes from the healthy (${L}_{W}$, ${L}_{B}$ and ${L}_{N}$) to non-healthy (${L}_{W}^{+}$, ${L}_{B}^{+}$ and ${L}_{N}^{+}$) condition can be modelled as follows.

All differences in non-healthy liver tissue fractions correspond to differences in ${L}_{W}$, ${L}_{B}$ or ${L}_{N}$ compartments or a combination of these. By defining changes in the non-water compartment ${\Delta L}_{N}=k{\Delta L}_{W}$ (where *k* is the proportion of differences in tissue water fraction ${L}_{W}$ corresponding to differences in the non-water compartment ${L}_{N}$), the tissue water fraction in non-healthy livers as a function of the non-healthy free water fraction (${W}_{F}^{+}$) can be modelled as:

5 $$L_{W}^{\dag } = \frac{{W_{F}^{\dag } \left( {L_{B} + L_{W} - kL_{W} } \right)}}{{1 - kW_{F}^{\dag } }}$$

In this model, *k* = 1 represents the case where all differences in tissue water fraction correspond to differences in the non-water fraction (${\Delta L}_{N}={\Delta L}_{W}$) and *k* = 0 represents the case where all differences in tissue water fraction correspond to differences in bound-water (${\Delta L}_{N}=0$). See Supplementary Material for full derivation.

As the source of free water variation in fibrotic patients is not fully established, $k$ remains an unknown parameter. In this study, the relationship between tissue water content and T_1_ for varying *k* was first modelled to assess the predictive capabilities of T_1_ measurements (Fig. 2a). Following this, the patient specific T_1_ was used to estimate ${W}_{F}^{+}$ according to Eq. 4 and then used to estimate ${L}_{W}^{+}$ from Eq. 5. MR derived tissue mass fat fraction (TMFF_MR_) was then estimated using Eq. 3 for varying values of the tissue-water-compartment proportionality constant *k* (0, 0.2, 0.4, 0.6, 0.8 and 1).

### Statistical analysis

All analysis was performed using SPSS software (version 22, IBM, Inc., Chicago, IL). Results are given as mean ± standard deviation. For the purposes of comparison, patients were grouped into groups of mild to no fibrotic (no bridging fibrosis, F0–F2) and severe fibrotic (bridging fibrosis and cirrhosis, F3–F4) and then linear regression was used to compare different methods of fat fraction analysis for each group.

The median and range of PDFF at each steatosis grading for none/mild *vs* moderate/severe fibrosis and none/mild inflammation *vs* moderate/severe inflammation were represented as box plots, with mild outlier defined as 1st quartile – 1.5 IQR or 3rd quartile + 1.5 IQR and extreme outlier defined as 1st quartile – 3 IQR or 3rd quartile + 3 IQR. The difference between mild and severe fibrosis PDFF test with significance set at *p* < 0.05.

## Results

The demographics of the group are outlined in Table 1. Figure 1 shows example spectra from one patient demonstrating T_2_ relaxation across the echo times.

**Table 1:** Demographics of patients in study (demographics of patients shown in parenthesis)

	Total	Male	Female
Number	110	85 (77)	25 (24)
Age (y) mean ± SD	50 ± 11	51 ± 12	49 ± 11
*Steatosis grade*			
0; <5% hepatocytes	27 (25)	21	6
1; >5–33% hepatocytes	44 (40)	39	5
2; >33–66% hepatocytes	19 (17)	16	3
3; >66% hepatocytes	20 (18)	9	11
*Inflammation*			
None/mild	66 (60)	50	16
Moderate	24 (22)	21	3
Severe	20 (18)	14	6
*Fibrosis score*			
None (F0)	24 (22)	16	8
Perisinusoidal or Periportal (F1)	30 (27)	22	8
Perisinusoidal and Periportal (F2)	24 (22)	20	4
Bridging (F3)	21 (19)	18	3
Cirrhosis (F4)	12 (11)	9	3
*Aetiology*			
Alcoholic liver disease	15 (14)	12	3
Hemochromatosis	3 (3)	3	0
Hepatitis B and C	22 (20)	20	2
Non-alcoholic fatty liver	62 (56)	46	16
Normal	6 (5)	3	3
Other	2 (2)	1	1

Simulations of the effect of tissue water content on PDFF and the observed T_1_ on tissue water estimates are shown in Fig. 2a and b. As tissue water content increases, the slope of the linear fit of PDFF against TMFF decreases.

**Fig. 3:**
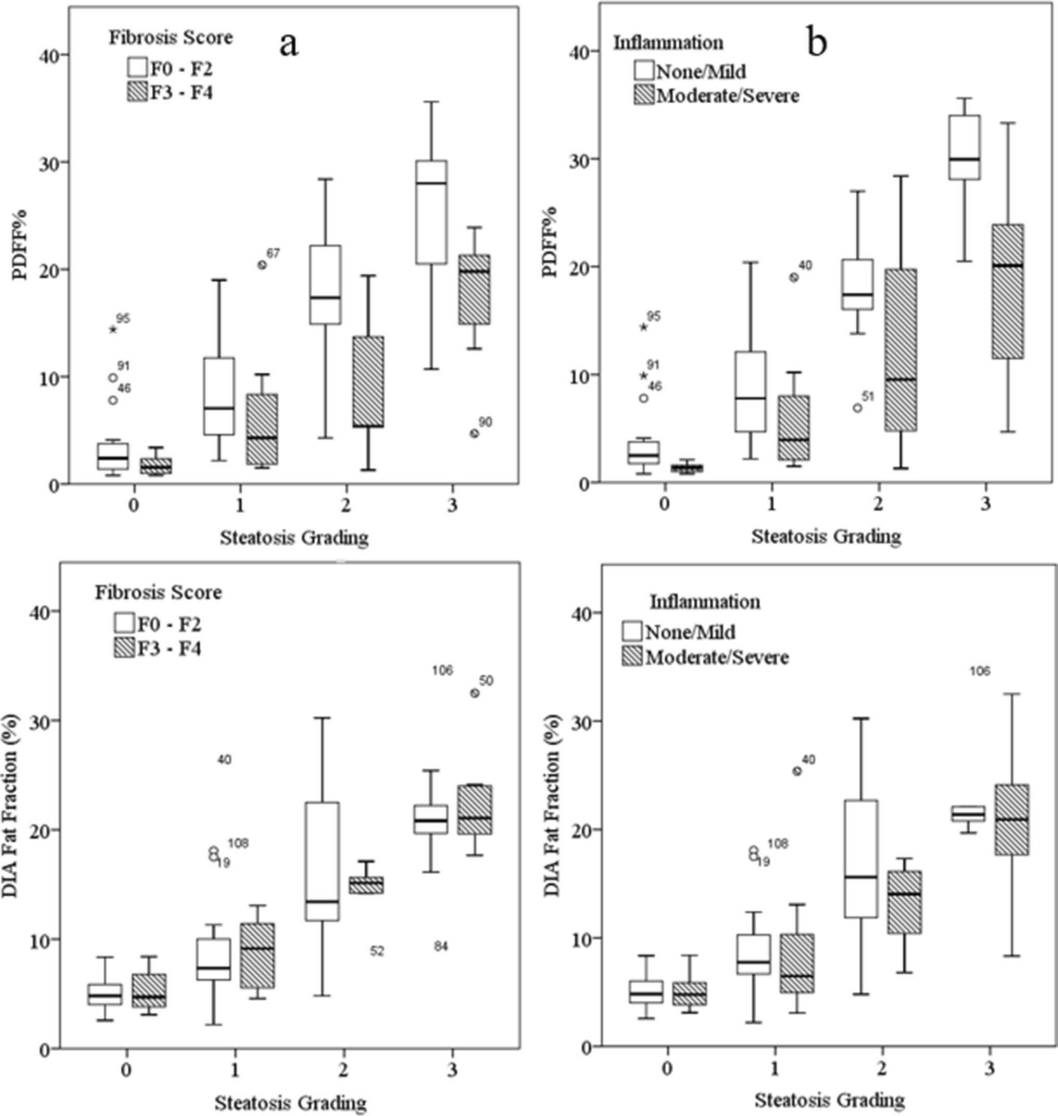
Box plot of MRS fat fraction (PDFF) at each steatosis grading split on fibrosis score (**a**) and inflammation (**b**) as labelled on graph. Mild outlier (unfilled circle) and extreme outliers (filled asterisk) are also shown with patient number attached

Mean DIAFF values were 5 ± 2%, 8 ± 4%, 15 ± 6% and 22 ± 5% and average PDFF values were 3 ± 3%, 7 ± 5%, 15 ± 8% and 22 ± 9% for VA steatosis gradings of 0, 1, 2 and 3, respectively. Figure 3 shows the variation in PDFF and DIAFF with steatosis grading when split on fibrosis score (F0–F2 vs F3–F4) and inflammation level (none/mild vs moderate/severe). There were significant differences in mean PDFF values between the F0–F2 and F3–F4 groups at steatosis grades 2 (*P* ≤ 0.05) and 3 (*P* < 0.005), and significant difference in mean PDFF values between none/mild and moderate/severe inflammation groups at steatosis grades 0 (*P* ≤ 0.005), 1 (*P* ≤ 0.05) and 3 (*P* < 0.005). The percentage difference in estimated values of PDFF in the F0–F2 *vs* F3–F4 groups was 50, 26, 50 and 30% for steatosis grades 0–3, respectively. There were no significant differences in mean DIAFF estimates between fibrosis groups F0–F2 *vs* F3–F4 or inflammation groups none/mild vs moderate/severe for any of the steatosis gradings.

**Fig. 4:**
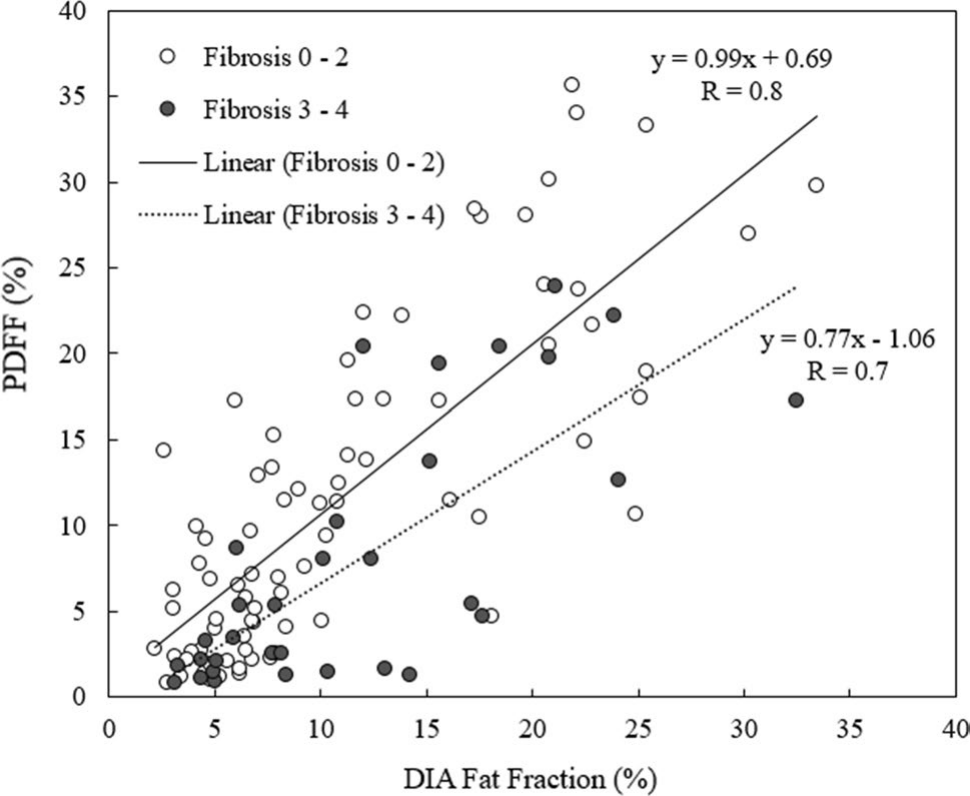
Plot of MRS proton density fat fraction (PDFF) against morphometry fat fraction split on low fibrosis grading (F0–F2) and high fibrosis grading (F3–F4). Linear regression fits are also displayed along with equations of fit, showing that in fibrotic livers PDFF estimates are lower than DIA fat fraction estimates

Both DIAFF and PDFF correlated significantly with VA of fat fractions (DIAFF: R = 0.83, *P* < 0.001; PDFF: R = 0.76, *P* < 0.001) with a slope of 0.23 and 0.25 for PDFF and DIAFF, respectively. Figure 4 shows a plot of PDFF against DIAFF with linear fits superimposed for the F0–F2 group (slope = 0.99) and the F3–F4 group (slope = 0.77) separately.

The T_1_ values for each fibrosis grading and the estimated liver tissue water content (based on Eqs. 3 – 5) for varying proportionality constant k are shown in Table 2. The average T_1_ for the F0–F2 and F3–F4 groups was 641 ± 39 ms and 711 ± 78 ms, respectively.

**Table 2:** T1 values at each fibrosis stage and the calculated tissue was content (%) for varying proportionality constant *k* (the proportion of tissue water increase coming from the non-water compartment)

Fibrosis score (F0–F4)	T_1_ ms	Estimated tissue free water content (%) for varying proportionality constant $k$					
		0	0.2	0.4	0.6	0.8	1
0	632 (37)	71 (2)	70 (2)	70 (2)	71 (3)	69 (4)	69 (8)
1	643 (35)	71 (1)	71 (2)	71 (2)	71 (3)	71 (4)	71 (8)
2	649 (44)	71 (2)	71 (2)	71 (3)	71 (3)	72 (5)	73 (10)
3	690 (70)	73 (2)	73 (3)	73 (4)	74 (5)	76 (8)	84 (19)
4	752 (80)	75 (2)	75 (3)	77 (4)	79 (5)	83 (8)	100 (24)

Table 3 summarizes the relationship between estimated TMFF_DIA_ and TMFF_MR_ for patients in the F0–F2 and F3–F4 groups for varying values of the theoretical proportionality constant $k$. The slope of the F0–F2 group stays close to unity, whereas the F3–F4 group increases from 0.79 to 0.84 for $k$ values between 0 and 1.

**Table 3:** The linear regression gradient and Pearson correlation coefficient in fibrosis groups F0–F2 and F3–F4 for correlations between digital image analysis (DIA) fat fraction and MRS calculated tissue mass fat fraction (TMFF) for varying proportionality constant, k (the proportion of tissue water increase coming from the non-water compartment)

$k$	Linear regression of correlation ($TMF{F}_{MR}$ v $TMF{F}_{DIA}$)			
	Slope		Pearson correlation coefficient	
	F0–F2	F3–F4	F0–F2	F3–F4
0	0.9	0.7	0.79	0.74
0.2	0.9	0.7	0.80	0.74
0.4	0.9	0.7	0.80	0.75
0.6	1.0	0.8	0.80	0.75
0.8	1.0	0.8	0.80	0.76
1	1.0	0.9	0.81	0.77

## Discussion

This study found a significant correlation between fat fractions measured using MRS, DIA and VA as expected. However, VA estimated up to four times greater percentage steatosis than DIA or MRS as previously reported [25, 26]. In contrast, the slope of correlation between PDFF and DIAFF was close to unity in healthy participants. Previous studies have reported similar strong correlations between DIA and VA assessments of the fat content from liver biopsies [32] and have found significant associations between these measurements and biochemical analysis [33]. Crucially, DIA provides an automated objective measure unlike VA.

The lower estimate of fat fraction using DIA and MRS compared with VA is expected given the difference in what is being measured. VA estimates the percentage of hepatocytes containing fat whereas DIA measures the percentage of fat relative to all tissue including nuclei, inflammatory cells, or scar tissue and MRS considers the percentage of fat relative to MR visible water. Previous studies have shown that VA provides excellent agreement across multiple pathologists [32], and can be combined with the simultaneous assessment of other relevant aspects of liver pathology. However both MRS and DIA provide objective measurements which agree well with each other and with biochemical analysis of hepatic fat in livers with mild to no fibrosis [14, 34]. This is required, for example, in experimental medicine studies of lifestyle and pharmacological interventions that monitor small changes in liver fat fraction over time [18], often in people expected to have low fat fractions.

However, PDFF was found to underestimate the fat content relative to VA more in fibrotic than non-fibrotic livers, similar to the result of McPherson et al. [24]. In the present study DIA measurements were also obtained and indicated a similar underestimation as PDFF in fibrosis (Fig. 4), suggesting that the previously reported differences between MR and VA in fibrosis cannot simply be attributed to errors in VA assessment.

There is some disagreement in the literature regarding the impact of fibrosis on PDFF measurements, though this may be due to differences in patient cohorts. Studies that found fibrosis not to be a confounding factor had a much smaller proportion of patients in the F2–F3 group (25% [26] and 23% [25] compared with 41% in our study, 34% in McPherson et al. [24] and 28% in Hajek et al. [15]*.*) and also less with severe inflammation (0% [26] and 4% [25] compared with 18% in the present study and 15% in McPherson et al*.*). In the present study, inflammation was also found to have a significant effect on PDFF across the steatosis grades; McPherson et al*.* did not find inflammation to be a confounding factor, but they only tested this in patients with mild to no fibrosis. It is almost impossible to separate the impact of inflammation from fibrosis as there are correlations between both conditions, and in the present study there were only 2 patients in the moderate/severe inflammation group who had no fibrosis (4 in F1).

In this study, changes in tissue water content were explored as a potential source of the discrepancy between PDFF and DIAFF in fibrotic patients. It was hypothesized that the previously noted increases in MR visible water content in fibrosis [23, 35, 36] act as a confounder in the MR measurements, resulting in decreased estimates of fat fraction compared with tissue fat fractions. Models of PDFF values for varying tissue mass fat fractions, as shown in Fig. 2a, demonstrate a progressive underestimation of fat fraction using MR techniques as the tissue water content increases from healthy liver values (~ 0.7). However, the underestimation found in fibrotic patients in this study appears to be even greater than the modelled data. For example, the mean PDFF at the greatest steatosis grading was 24 ± 9% in fibrotic grades 0–2 and 17 ± 6% in fibrotic grades 3–4. According to the modelled data, a tissue mass fat fraction of 25% would only be underestimated as a PDFF of 19% at the maximum free water tissue content (${L}_{F}=1$), although this does assume that fibrotic grades 0–2 have a healthy free water tissue content. This suggests that there may be other factors effecting the PDFF measurement in fibrotic patients.

By modelling the relationship between tissue water content and T_1_ (Fig. 2b), the patient-specific T_1_ measurements were investigated as an independent indicator of tissue water content in an attempt to correct MR derived fat fraction measures (Table 2). Although in this study the data were acquired using a 1.5 T system, this model will also apply at greater field strengths (e.g. 3 T) which may provide a greater range of correction factors due to the larger T_1_ values. The source of change in tissue water in the non-healthy compared to healthy tissue (*k*) had a big impact on these effects in the model, with *k* = 1 (representing all differences in water corresponding to differences in non-water compartments) leading to large changes in tissue water content for small changes in T_1_. Further work is needed to characterize this unknown parameter.

Using patient specific T_1_ to estimate tissue water content, this study was able to derive an MR based estimate of tissue mass fat fraction which accounted for potential changes in fibrotic tissue. Table 2 shows an improvement in correlations between corrected MR fat fraction TMFF_MR_ and TMFF_DIA_ in fibrotic patients. As may be expected from the hypothesis, the slope of relationship in non-fibrotic participants remained relatively unchanged which is a result of the fact that the T_1_ values in this group are closer to healthy tissue T_1_. This can be seen by noting the point of convergence in Fig. 1b where varying k has little impact on ${L}_{F}$. By contrast, in the fibrotic group the slope of relationship moved towards unity for increasing k, again indicating that T_1_ is providing an estimation of tissue water content.

However, despite improvements in the correlations between DIA and MR derived fat fraction using tissue water content estimates, there remained an underestimation in the fibrotic patient group. Even assuming the maximum value of k, i.e. all fractional differences in tissue water content in non-healthy compared to healthy livers is from non-water compartments, TMFF_MR_ remained 90% of TMFF_DIA_*.* This suggests that, whilst tissue water content may contribute to the underestimation of fat fraction in fibrosis, there are other factors also to consider.

Other potential sources of underestimation in MR measurements may be the effects of iron concentration, which tends to reduce T_2_ and T_2_* via diffusion and field inhomogeneity [37]. However, the MRS protocol used in this study was acquired using spin echoes which removes field inhomogeneity effects and signals were corrected for T_2_ relaxation. It is also possible that T_1_ values may be reduced in the presence of iron. However, the cohort in this study where predominantly low iron (83% in 0–1 on histology) and there was no correlation between T_1_ and iron levels (*R*^2^ = 0.014, *P* = 0.226).

Alternatively, potential errors in histological analysis should not be ruled out. Although used as the clinical standard, both VA and DIA rely on H&E staining where swollen water vacuoles can remain unstained and lead to difficulties in distinguishing between fat and water regions. This would lead to an overestimation of fat content in fibrotic tissue with swollen vacuoles which would further affect the discrepancy with MR measurements. Previous studies have validated MR measurements using biochemical assessments of fat content [14] which would seem to give the most accurate measurement of tissue fat fraction, indicating that MR may provide a more objective estimation (although the previous discussion of PDFF vs TMFF should be considered). To explore this further, Oil Red-O staining could be compared with H&E, which binds only to the fat molecules and has previously been shown to provide very accurate measurements of fat [38].

PDFF remains a key marker of liver disease which benefits from being objective and non-invasive. Given the importance of liver fat content in the early and pre-disease stages of liver disease and metabolic disorders, and the strong correlation between PDFF and tissue mass fat fraction in healthy livers, this MR measurement provides a powerful non-invasive methodology for use clinically and in experimental medicine. However, this study has demonstrated potential MR confounders that may lead to discrepancies between PDFF and TMFF in the diseased case. Since liver lipid content acts as an indicator of non-liver related clinical outcomes, for instance, in type 2 diabetes, and is used to stratify patients into pathways of management, accurate determination of tissue fat fraction across disease states may be crucial. As such, clinicians would benefit from understanding the differences between PDFF and TMFF. Recently there have been a number of commercial packages offering liver fat fraction estimates from CSE-MRI acquisition as part of a range of clinically relevant measures. These products should be careful to indicate that PDFF values are an MR surrogate of true tissue fat fraction and should also consider including MR estimates of TMFF to provide results comparable to histological data especially in fibrotic livers.

The findings of this study are most relevant in experiment design, for example, when investigating novel therapeutic interventions in fibrosis or cirrhosis or when comparing fibrotic and non-fibrotic patient groups. Such studies should recognize the potential confounds in MR estimates and give careful attention when planning MR acquisition and during analysis. Recent work has developed simultaneous fat fraction, T_1_ and T_2_^*^ measurements [39] which could be used to acquire multiple quantitative measures useful for conversion to TMFF.

There were some limitations in this study. First, the MR signal was localized to a region of interest which may not have matched the region that was biopsied. Spectra were acquired from a similar region to the biopsy, and previous work has suggested consistent results within respective hepatic lobes [40] with only small variations across the whole liver [41], but this is likely to have contributed to the scatter in the data. Secondly, PRESS MRS was used rather than STEAM or CSE-MRI which has previously been shown to potentially effect final fat fraction calculations [20]. PRESS was used to reduce motion artifacts from respiratory triggered acquisition. However, any effects of using PRESS will be consistent across the data and do not impact the underestimations observed in fibrosis. Finally, some assumptions about physical parameters were made to estimate a corrected TMFF from MR and DIA measurements. These are outlined in the methods section. Although this work was carried out using localized spectroscopy data the issues identified are fundamental to all MR methods (including gradient echo imaging approaches), although the T_1_ and T_2_ weighting will depend on readout approach.

In conclusion, this study has confirmed previous findings that PDFF underestimates fat fraction compared to histological measures in fibrotic patients. Modelling demonstrated the importance of tissue water and T_1_ on PDFF, and researchers should account for the impact of this where appropriate. Estimates of tissue water content may be derived from independent T_1_ measurements and used to improve correlations between MR and histological measurements, but do not fully explain the discrepancies. Further work is needed to establish the accuracy of true tissue mass fat fraction measurements in both MR and H&E staining.

## Supplementary Information

Below is the link to the electronic supplementary material.

Supplementary file1 (DOCX 22 KB)

## Data Availability

The raw/processed data required to reproduce the above findings cannot be shared at this time as the data also forms part of an ongoing study. Please contact the corrosponding author for futher access.

## Abbreviations

MRS: Magnetic resonance spectroscopy
PDF: Proton density fat fraction
VA: Visual assessment
DIA: Digital image analysis
DIAFF: DIA fat fraction
NASH: Non-alcoholic steatohepatitis
NAFLD: Non-alcoholic fatty liver disease
MR: Magnetic resonance
MRI: Magnetic resonance imaging
TMFF: Tissue mass fat fraction
PRESS: Point resolved spectroscopy
TE: Echo time
SNR: Signal to noise ratio
H&E: Hematoxylin and eosin
CRN: Clinical research network
