# Supplementary material for: Comparing magnetic resonance liver fat fraction measurements with histology in fibrosis: the difference between proton density fat fraction and tissue mass fat fraction
Source: MAGMA. 2022 Dec 20;36(4):553–63. doi: 10.1007/s10334-022-01052-0 (PMC10468948; doi:10.1007/s10334-022-01052-0)
Supplement: Supplementary file 1 — Supplementary file1 (DOCX 22 KB) [file 10334_2022_1052_MOESM1_ESM.docx]

**Supplementary Material**

*Calculation of tissue water content from* $T_{1}$

Begin by using a three compartment liver tissue model where $L_{W}$, $L_{B}$ and $L_{N}$ are the liver tissue fractions of free water, bound water, and non-water tissue compartments respectively:

$$L_{W}+L_{B}+L_{N}=1$$

Equation 1

The fraction of free and bound water is therefore

$W_{F}=L_{W}/(L_{W}+L_{B})$ and $W_{B}=L_{B}/(L_{W}+L_{B})$

Equations 2a and b

Where the subscript $F$ and $B$ denotes free and bound water respectively; and $W_{F}+W_{B}=1$.

Compared to healthy livers, non-healthy livers will differ in tissue fraction such that:

$$L_{W}=L_{W}+\Delta L_{W}$$

$$L_{B}^{\dagger}=L_{B}-(1-k)\Delta L_{W}$$

$L_{N}^{\dagger}=L_{N}-k{\Delta L}_{W}$

Equations 3a, b and c

Where the superscript † denotes non-healthy liver tissue; $\Delta L_{W}$ is the difference in free water tissue fraction in the non-healthy compared to healthy tissue; and $k$ is the fraction of change coming from the non-water compartment $L_{N}$ (i.e. ${\Delta L}_{N}=k{\Delta L}_{W}$)

Substituting this into Equation 1 gives:

$$L_{W}^{\dagger}+L_{B}+L_{N}-\Delta L_{W}=1$$

Equation 4

Now substitute in Equation 2a:

$$W_{F}^{\dagger}\left( L_{W}^{\dagger}+L_{B}^{\dagger} \right)+L_{B}+L_{N}-\Delta L_{W}=1$$

Now use equation 3b:

$$W_{F}^{\dagger}L_{W}^{\dagger}+W_{F}^{\dagger}(L_{B}-\left( 1-k \right)\Delta L_{W})+L_{B}+L_{N}-\Delta L_{W}=1$$

Substitute in equation 4 and simplify:

$$W_{F}^{\dagger}L_{W}^{\dagger}+W_{F}^{\dagger}L_{B}-W_{F}^{\dagger}L_{W}^{\dagger}+W_{F}^{\dagger}L_{W}+kW_{F}^{\dagger}L_{W}^{\dagger}-kW_{F}^{\dagger}L_{W}+L_{B}+L_{N}-L_{W}^{\dagger}+L_{W}=1$$

$$W_{F}^{\dagger}L_{B}+W_{F}^{\dagger}L_{W}+kW_{F}^{\dagger}L_{W}^{\dagger}-kW_{F}^{\dagger}L_{W}+L_{B}+L_{N}-L_{W}^{\dagger}+L_{W}=1$$

Rearrange this to give:

$$L_{W}^{\dagger}-kW_{F}^{\dagger}L_{W}^{\dagger}=W_{F}^{\dagger}L_{B}+W_{F}^{\dagger}L_{W}-kW_{F}^{\dagger}L_{W}+L_{B}+L_{N}+L_{W}-1$$

And include $L_{B}+L_{N}+L_{W}=1$ to simplify:

$$L_{W}^{\dagger}\left( 1-kW_{F}^{\dagger} \right)=W_{F}^{\dagger}L_{B}+W_{F}^{\dagger}L_{W}-kW_{F}^{\dagger}L_{W}$$

$$L_{W}^{\dagger}=\frac{W_{F}^{\dagger}\left( L_{B}+L_{W}-kL_{W} \right)}{1-kW_{F}^{\dagger}}$$
